# Blood-Letting in Disease

**Published:** 1880-10

**Authors:** Harvey L. Byrd

**Affiliations:** Professor of Obstetrics in Washington University of Baltimore, Md.


ARTICLE IV.

### BLOOD-LETTING IN DISEASE.

BY HARVEY L. BYRD, M. D.
Professor of Obstetrics in Washington University of Baltimore, Md.

It may seem strange to some that it should be thought necessary by any one to call the attention of the profession, at the present day, to so ancient a remedy as blood-letting, and particularly to claim for it the highest rank as a therapeutical agent. And yet the writer feels that it should be done in the interests of humanity, if for no other motive or cause. It constitutes no part of his purpose, however, to attempt a history of the remedy or to epitomize even its leading facts and great achievemnts in the past, further than may be necessary to a presentation of the advantages it is capable of affording in the treatment of disease at the present time. It is proper to remark, in passing, that it shared the confidence, to a greater degree, of those physicians whose names have come down to us as the great intellectual luminaries of the ages in which they lived than any other remedy whatsoever. Though shrouded in the impenetrable darkness of the unknown past, the use of blood-letting in the treatment of disease was, doubtless, like many other agents, the result of accident, and at a period nearly, if if not quite, coeval with the creation of the tribes of the higher order or varieties of the human race.

In looking down the shadowy corridors of the past, we find it occupying high position in the healing art. HIPPOCRATES places it, so to speak, at the top of the list of therapeutic agents, and speaks of it as a remedy familiarly known and used, or practiced for the relief of disease by others as well as himself. ASCLEPIADES, a Greek physician of great renown, who practiced in Rome more than a hundred years before the Christian era, resorted to blood-letting to a considerable extent. He also used scarification with cupping in the treatment of certain morbid condition. ARETÆUS was not only an advocate of the several methods of abstracting blood in vogue at the present time, but was among the earliest if not the first, to practice arteriotomy. In speaking of him HALLER says: "*Arterias incidit ante Galenum, in temporibus et ad aures alibique.*" Then the methodic sect, CELSUS and GALEN, were all the warm advocates of blood-letting. The Arabian physicians, of whom AVICENNA was the most prominent and justly distinguished, adopted the practice of the Greeks in all essential respects. The Egyptians resorted to blood-letting in all its varieties. PARACELSUS notwithstanding his elixirs, used blood-letting quite extensively in the treatment of disease.

With few exceptions, including the periods from Paracelsus, and revival of learning in the fifteenth century, on down to the first quarter of the present century, blood-letting has had for its champions and supporters the brightest intellects of the profession.

The second quarter of the nineteenth century has had fewer prominent advocates for the use of our remedy than probably any equal period of time in its history; and that, too, when neither superstition nor religious fanaticism can be regarded as having exerted any influence on the practice of the remedy. Why, then, has bleeding been so little resorted to within this period? Is the type of disease more *asthenic* than formerly? Or are there remedies of *equal* efficacy that have been substituted for sanguinous depletion? As the writer is voluntarily on the stand before the public, it is proper he should furnish such testimony as he may be able from long and varied experience, and in doing so, he flatters himself that satisfactory answers will be given to some of the above interrogatories.

In offering his experience with the lancet, no arrogation of superior sagacity, or claims of a better acquaintance than others with morbid phenomena is attempted, but simply a statement of some of the beneficial effects witnessed from its use in a great number of cases, in a practice extending over a period of nearly thirty years, between the thirtieth and thirty-ninth degrees of north latitude. No hesitation is experienced in pronouncing the lancet *sans pareil* in the treatment of a large number of the diseases to which flesh is heir, in several of which *fashion* has unfortunately inhibited its use in these latter days. Even age and experience in practice being forced in such cases to yield to the inexorable demand of the fickle goddess. Where the following conditions and circumstances receive, as they always should, careful consideration, before an important remedy is administered for the relief of morbid action, viz: Race, age, sex, temperament, previous and present condition of the patient, bleeding, will, when called for, yield results of a more highly satisfactory character than *any other* remedial agent!

The effects of sanguineous depletion are marked, and unmistakable in the production of antiphlogistic, sedative, antispasmodic, and alterative impressions on the systems. It will thus be seen in what a large number of cases it may be resorted to with great advantage in their treatment.

In *all* inflammatory diseases, when properly timed under a judicious appreciation of the above-named conditions, the writer of this article has no hesitancy in denominating the lancet the most scientific, as it certainly is the most efficient and valuable, agent in our present list of therapeutical resources. If there is one single fact in the whole range of our professional observation or experience that may be certainly regarded as established, and beyond the possibility of contradiction or satisfactory refutation, it is the utility, nay more, the indispensable necessity for sanguineous evacuation, in the prompt and scientific treatment of inflammation! Just here we are strongly tempted to disregard our intention when commencing this article, and go somewhat into detail concerning our experience with the lancet in the several forms of inflammatory disease. But a moment's reflection assures us that a volume might easily be filled with such cases, much less, all the pages of the REPORTER could they be devoted to us exclusively, and we must therefore hasten to the mention of other conditions, in which the most gratifying results have been experienced from blood-letting.

In cerebritis, arachnitis, apoplexy, congestion, or extravasation, in the eclampsia, and in fact, in almost every form of cerebral disease, the lancet is the most important remedy at our command. In certain conditions of the brain it is not only simply *indispensable to the successful termination of the case, but often the only remedy necessary* to be used at all. It is often not only the best, but frequently the *only* remedy capable of relieving the obstinate constipation resulting from inflammation of the muscular coat of the intestines. In hernia, in the rigidity and spasm of the os and cervix uteri, in labor, to say nothing of other conditions of muscular spasm, and contractions beyond the anæsthetic reach of chloroform, and, lastly, its alterative action, is quite manifest in promoting the absorption of fluid in various cavities and situations of the body.

In conclusion, it is contended that *the necessity for the use of the lancet is as great at the present time as it ever was in the past*, and that the type of disease has undergone no such change as to render the abstraction of blood unnecessary or improper in the successful management of all cases attended with a full tense and quick pulse.

. This subject is one of great moment, yet capable of easy comprehension and satisfactory demonstration, and as such the careful attention of the junior members of the profession is respectfully and earnestly invited to it. We feel warranted, from our long experience, in saying that any intelligent physician, who will resort to the lancet in a few cases calling for its use, will not be likely to lay it aside afterwards, though he should encounter the jeers of some or the *quasi* assent only of others, who might entertain from early training, or other cause, prejudice against its use. And such we feel assured will be the testimony of all who have not sacrificed the lancet on the alter of fashion. The aid of the profession is invoked for the purpose of placing once more on the stage of usefulness a remedy which has played so important a part in the almost entire history of our therapeutics.

